# Comparison of mathematical models to predict glass transition temperature of rice (cultivar IRGA 424) measured by dynamic mechanical analysis

**DOI:** 10.1002/fsn3.785

**Published:** 2018-10-29

**Authors:** M. Mercedes Bertotto, Analía Gastón, María J. Rodríguez Batiller, Pablo Calello

**Affiliations:** ^1^ Faculty of Engineering University of Buenos Aires Buenos Aires Argentina; ^2^ SENASA National Service of Agrifood Safety and Quality Buenos Aires Argentina; ^3^ Faculty of Exact Sciences, Engineering and Land Surveying CIC‐UNR IFIR (CONICET/UNR) Rosario Argentina; ^4^ National Council of Scientific and Technical Research (CONICET) Institute of Technology in Polymers and Nanotechnology (ITPN) Faculty of Engineering University of Buenos Aires Buenos Aires Argentina

**Keywords:** dynamic mechanical analysis, food processing, glass transition, mathematical modeling

## Abstract

Dynamic mechanical analysis (DMA) was applied to measure the Tg of rice IRGA 424 at different moisture content values (9.3%–22.3% wet basis). To conduct temperature sweeps, the samples were heated at a rate of 2°C/min from 20 to 120°C keeping frequency to 1 Hz. Tg was measured both from the *E*″ peak temperature (Tg_midpoint_) and from the *tan (*δ*)* peak temperature (Tg_endset_). Tg_midpoint_ and Tg_endset_ increased from 31.8 to 86.6°C and 42.1 to 104.7°C, respectively, as moisture content decreased from 22.3 to 9.3%. Six models were tested for their ability to predict Tg as a function of the moisture content. As all residuals were normally distributed and homoskedastic, standard metrics were used to assess the fitted models. Goodness of fit by these models was established by comparing the coefficient of determination (*R* ^2^), standard error of the estimate (SEE), and mean relative deviation (MRD). The Gordon–Taylor linearized equation was the most accurate in predicting Tg. To predict Tg from the moisture content of the rice samples, a new expression was proposed. For the conditions considered in this work, the developed equation satisfactorily predicts the Tg of rice IRGA 424 without needing prior linearization.

## 1 INTRODUCTION

Among cereals, rice (*Oryza Sativa* L.) is considered to be the staple food for nearly one half of the world's population (Grossa & Zhaob, 2014). Rice is usually harvested with high moisture content (MC). As this is not suitable for storage and preservation, it is necessary to resort to a drying process. However, if this operation is not carried out under controlled conditions, MC gradients (MCGs) are created in the kernels, which may cause fissures (Ban, 1971; Craufurd, 1963; Kunze, 1979; Nguyen & Kunze, 1984; Sarker & Kunze, 1996; Sharma & Kunze, 1982; Yang, Zhang, & Jia, 2005). In order to reduce the MCGs, rice is usually held between passes in a multiphase drying process. This practice is known as tempering, which allows moisture to migrate from the core to the outer layers of the kernel.

Fissure formation is a major concern during rice drying because fissured kernels are not only more susceptible to breakage in the milling process, but also affect the functional properties of milled rice. Understanding the effect of MCGs, as well as temperature gradients, on rice fissure formation is important in controlling and optimizing drying and tempering conditions. This can be explained taking the glass transition temperature (Tg) of rice into account (Cnossen, Siebenmorgen, Yang, & Bautista, 2001; Cnossen, Siebenmorgen, & Yang, 2002; Cnossen, Jimenez, & Siebenmorgen, 2003; Cnossen, Siebenmorgen, Yang, & Bautista, 2001; Ghasem, Sadeghi & Miree, 1960; Iguaz, Rodriguez, & Virseda, 2006; Yang, Jia, Siebenmorgen, Howell, & Cnossen, 2002; Yang & Jia, 2003, 2004; Yang et al., 2005). Tg can be defined as the temperature at which an amorphous system changes from the glassy to the rubbery state and vice versa. In amorphous systems, Tg depends on temperature, time (or frequency), and composition (Slade & Levine, 1995). Whether a rice kernel is above or below Tg has been shown to significantly affect its physical properties as well as drying rate and fissure initiation in the rice kernel (Cnossen & Siebenmorgen, 1999; Cnossen et al., 2002; Perdon, Siebenmorgen, & Mauromoustakos, 2000).

Drying and tempering rice above the glass transition temperature is more effective in preserving its quality, because regions with different mechanical properties are not generated, and there is not sufficient stress to cause fissuring in the kernel (Cnossen et al., 2002; Schluterman & Siebenmorgen, 2007; Yang & Jia, 2003; Zhang, Yang, & Jia, 2003).

It is worth mentioning that the response of rice varieties to drying operations differs, depending primarily on their origin and genetic factors. To the best of our knowledge, no information regarding relevant parameters such as Tg has been found for IRGA 424, the long‐grain rice variety most widely cultivated in the Argentine Republic. For this reason, information on Tg for this variety is urgently needed.

In this study, Tg of IRGA 424 as a function of MC was determined by dynamic mechanical analysis (DMA). DMA works by applying a sinusoidal deformation to a sample of known geometry. The sample can be subjected to a controlled stress or strain. For a known stress, the sample will then deform a certain amount. The nature of this response may be used to determine the elastic and viscous properties of the material (storage modulus and loss modulus, respectively), and this can be very sensitive to Tg as the material rapidly becomes more flexible at the transition temperature. A third parameter, *tan (*δ*)*, is defined as the ratio of the loss modulus (*E″*) to the storage modulus (*E′*) and represents the relative contribution of the viscous vs. elastic properties. However, materials do not have a single glass transition, and Tg is defined more as a range of temperatures within which the material undergoes drastic changes in its thermomechanical properties (PerkinElmer, Inc., 2008).

After scanning the sample under test, Tg can be evaluated according to three criteria (Turi, 1997):

(Tg_onset_): inflection point temperature of the *E′* curve.
(Tg_midpoint_): *E″* peak temperature.
(Tg_endset_): *tan (*δ*)* peak temperature.

At Tg, the increase in molecular motion within the polymer results in a dramatic step decrease in *E′*, making DMA probably the most sensitive thermal technique for Tg determinations.

Tg of rice varieties of different origins has been evaluated by various research groups. Perdon et al. (2000) studied the effect of MC on Tg of individual kernels of Bengal (medium‐grain) and Cypress (long‐grain) brown rice. Sun, Yang, Siebenmorgen, Stelwagen, and Cnossen (2002) used thermomechanical analysis (TMA) and differential scanning calorimetry (DSC) to investigate the thermal transitions in Drew (long‐grain) rice kernels. Siebenmorgen, Yang, and Sun (2004) measured Tg of Drew and Bengal rice samples using DMA. Thuc, Fukai, Truong, and Bhandari (2010) applied a thermal mechanical compression test (TMCT) to measure the Tg of rice flour and individual rice kernels of YRM64 rice, an Australian genotype. More recently, Talab et al. (2012) used DSC to measure Tg of MR219, a rice variety from Malaysia. The Bengal cultivar was also studied for its phase transition characteristics by Nithya, Saravanan, Mohan, and Alagusundaram (2015).

While Tg can be measured by a variety of methods, it is a time‐consuming procedure. Hence, predicting rather than directly measuring Tg as a function of the MC of rice can be a powerful tool. Once the coefficients of the models have been fitted to the experimental data, the equations can be used to predict the Tg of other rice samples of the relevant variety. It was therefore necessary to select a mathematical model that best fits the data of the variety under investigation.

The objectives of this work were (a) to determine the glass transition temperatures of IRGA 424 as a function of moisture content using DMA; (b) to test five mathematical models for their ability to predict Tg as a function of the water content of the rice samples; and (c) to develop a new mathematical model to predict the Tg of rice directly as a function of moisture content.

## 2 EXPERIMENTAL PROCEDURE

### 2.1 Rice samples

The variety of long‐grain paddy rice IRGA 424 was used in this study. It was provided by the Faculty of Agricultural Science of the North‐East National University, Argentina. This material was received with a moisture content of 9%. The moisture content is expressed as a percentage of wet basis (w.b.) throughout this article unless stated otherwise.

### 2.2 Sample preparation

The defective, unshelled, or broken grains were manually separated and discarded. The sample was sieved to remove dust and plant remains. Paddy rice grains (100 g) were processed in a rice laboratory mill (Suzuki MT‐95, Suzuki S.A., San Pablo, Brazil). This equipment performed the dehulling of rice grains through grinding, producing a dehusked grain fraction known as brown rice (or unpolished rice).

The samples of brown rice, cultivar IRGA 424, were then moisturized in 30 sample flasks. Each sample of 50 g of rice was weighed, and the volume of water required to reach the desired final moisture content was added to the flask. The flasks were shaken and stored in the refrigerator for 7 days at a temperature of approximately 5°C. During this time, the water was absorbed by the grain mass, so that a uniform moisture distribution was obtained. As a result of this rehydration process, 24 samples were obtained corresponding to the eight moisture content levels, in triplicate: 10.21%, 13.12%, 14.48%, 15.59%, 17.37%, 18.15%, 19.78%, and 21.68%.

Samples of rice flour were obtained by milling the samples of hydrated rice. Grains (30 g) were processed in a Decalab^®^ laboratory knife mill. Later, the flour sample was sieved, separating the fraction below 177 μm. To do this, a sieve (ASTM No. 80s, Gilson Company, Inc.) and a vibrator (Zonytest EJR 2000) were used.

Flour moisture content was determined gravimetrically according to AACCI Method 44‐15.02. A small sample of the product was weighed and placed in a moisture dish. The sample was heated in a forced convection oven at 130°C for 1 hr and cooled to room temperature, and finally, the residue was reweighed. The moisture loss was then calculated.

The hydrated flour samples were packed into airtight jars and labeled according to their moisture content for glass transition temperature determination.

### 2.3 DMA measurement

The transitions of rice flour samples were determined by a dynamic mechanical analyzer (DMA, PerkinElmer 8000, 2014). DMA data were obtained over a temperature range of 20–120°C, on the deformation mode. The samples were heated at a rate of temperature increase of 2°C per minute. A low frequency of 1 (Hz) was used to minimize its effect on Tg. The geometry and mode of deformation of the sample used were “single cantilever bending”; the type of frequency control was simple (single frequency/single strain).

The force resolution was 0.002 N, and tan delta resolution was 0.00001. DMA equipment could be operated with a measuring range of ±1,000 μm in tension and compression, sensitivity of 10 N·m, and force of minimum of 0.002 N and maximum of ±10 N, with samples of up to 10 mm.

A material pocket held the sample, so it could be mounted in a DMA 8000 instrument. It was a stainless steel envelope that also helped to limit the slight loss of moisture that could be expected due to the increase in temperature during the tests. The material pocket did not have any relaxations or phase transitions over the temperature range of the instrument (PerkinElmer, Inc., 2011).

Using the DMA methodology explained above, Tg values of the 24 samples of hydrated rice were obtained. The selected moisture content range was from 9.3% to 22%, according to the postharvest conditions.

## 3 MATHEMATICAL MODELS FOR PREDICTION OF GLASS TRANSITION TEMPERATURES

Mathematical models for prediction of the Tg of rice samples are based on the free volume theory, the kinetic theory, and the thermodynamic theory. Although different in detail, all of them consider the additivity of basic properties.

### 3.1 Expressions for estimating Tg selected from literature

Gordon and Taylor equation is based on two basic assumptions: volume additivity and a linear change in volume with temperature:
  (1) $$T_{g}=\frac{w_{1}T_{g1}+kw_{2}T_{g2}}{w_{1}+kw_{2}},$$

where *w*_2_ = 1 − *w*_1_.

The subscripts 1, 2, and *m* denote component **1**, component **2**, and the mixture, respectively, and *w* is the weight fraction concentration in the mixture. The term *k* in Equation (1) is a parameter whose value depends on the change in thermal expansion coefficient of the components as they change from the glassy (amorphous) to the liquid (rubbery) form, during the glass transition. A common simplification is to let *k* be a curve‐fitting parameter (Ross, 2010).

Equation (1) was linearized for the parameter *k*:

(2) y=kx,

where

(3) $$y=w_{1}\left(T_{g}-T_{g1}\right),$$

(4) $$x=\left(1-w_{1}\right)\left(T_{g2}-T_{g}\right)$$

The Fox model, shown by Equation (5), describes a weighted‐average relationship between Tg and the mass fraction of the component. It is assumed that the product of Tg and the change in specific heat are identical for all components (Fox, 1956). This is the limiting case where *k *=* *1.

(5) $$\frac{1}{T_{g}}\approx \sum \frac{\omega_{i}}{T_{\mathrm{gi}}},$$

where Tg and Tg_*i*_ are the glass transition temperature of the mixture and of the components and ω_*i*_ is the mass fraction of component *i*.

Jenkel and Heusch (1953) proposed the Equation (6), which accounts for monotonic (all positive or all negative) deviations from the linear combination, where *k* is an empirical fitting parameter:
  (6) $$T_{g}=w_{1}T_{g1}+w_{2}T_{g2}+kw_{1}w_{2}$$
Kwei (1984) investigated polymer mixtures and proposed the following empirical expression:
  (7) $$T_{g}=\frac{w_{1}T_{g1}+w_{2}T_{g2}}{w_{1}+kw_{2}}+qw_{1}w_{2}$$

where *k* and *q* are fitted parameters, whose meaning is based on the intermolecular interactions between the components in the mixture.

The application of the aforementioned equations requires the knowledge of Tg of water (Tg_1_) as well as the value of glass transition of pure starch (Tg_2_). Forster, Hempenstall, and Rades (2002) have tabulated several values for Tg_1_ commonly used in the literature. These values ranged between −134 and −143°C. The currently used value of −136°C was taken as Tg_1_ in this study.

Tg_2_ was obtained by Sablani, Bruno, Kasapis, and Symaladevi (2009) using Gordon–Taylor equation, interpolating the glass line until solid content value is 1. These authors estimated a Tg_2_ value of 158.5°C.

### 3.2 A new mathematical expression

The following equation was derived from experimental findings of Tg of IRGA 424:

(8) $$T_{g}=a-1,000\frac{(e^{kw_{1}}-1)}{k}$$

where *a* and *k* are fitting parameters.

## 4 STATISTICAL ANALYSIS OF Tg DATA

Mean, standard deviation (SD), standard error of the mean (SEM), and coefficient of variation (CV) values were reported in all cases (Table 1). CV and SD are measures of relative and absolute dispersions, respectively. CV is dimensionless, while SD has the units of the variable quantified. The advantage of using the CV was that it could compare across different variables because they were measured on the same relative scale.

**Table 1:** Glass transition of IRGA 424 measured by differential mechanical analysis (DMA)

Moisture content %				Tg Midpoint				Tg Endset			
Mean	*SD*	*SEM*	CV	Mean	*SD*	*SEM*	CV	Mean	*SD*	*SEM*	CV
10.21	1.15	0.66	11.27	80.36	10.80	6.24	13.44	97.42	12.34	7.12	12.66
13.12	0.21	0.12	1.59	65.99	1.70	0.98	2.57	80.91	2.91	1.68	3.60
14.48	0.45	0.26	3.11	61.81	2.41	1.39	3.89	74.90	3.65	2.11	4.88
15.59	0.43	0.25	2.77	54.74	3.90	2.25	7.12	75.91	3.74	2.16	4.93
17.37	0.55	0.32	3.16	53.05	4.40	2.54	8.29	65.95	9.89	5.71	15.00
18.15	0.71	0.41	3.91	40.16	0.94	0.54	2.33	47.55	6.14	3.55	12.91
19.78	0.52	0.30	2.65	34.79	2.79	1.61	8.02	42.07	2.36	1.36	5.60
21.68	0.84	0.48	3.87	34.49	4.55	2.63	13.18	43.05	1.54	0.89	3.58

Note

$SD=\sqrt{\frac{\sum {\left(A-\overset{¯}{A}\right)}^{2}}{N}}\;\text{CV}=\frac{\mathrm{SD}}{\overset{¯}{A}}$ where *SEM*: standard error of the mean; *SD*: standard deviation; CV: variation coefficient; *A*: each value in the data set; $\overset{¯}{A}$: mean of all values in the data set; *N*: number of values in the data set.

Nonlinear regressions and data processing were performed using OriginPro version 8.0 (2003) and Microsoft Office Excel 2007 (Microsoft Corporation, Redmond, USA).

The aim of nonlinear fitting is to estimate the parameter values which best describe the data. The difference between the observed value of the dependent variable and the predicted value is called “residual.” The standard way of finding the best fit is to choose the parameter values that minimize the residuals. In this method, called “ordinary least squares (OLS),” the overall solution minimizes the sum of the squares of the residuals made in the results of every single equation.

OLS method can only be applied for nonlinear fitting of a model if its residuals are normally distributed and if they have homoskedasticity (meaning “same variance”).

To that end, two different tests assessing the *p*‐value were carried out in the Origin software program: Levene's test (Levene, 1960) and Shapiro–Wilk test (Shapiro & Wilk, 1965).

In order to assess the assumption of normality, Shapiro–Wilk tests were carried out for the residual data of each model. The null hypothesis for this test was that the data were normally distributed. If the chosen alpha level was 0.05 and the *p*‐value was less than 0.05, then the null hypothesis that the data were normally distributed was rejected. If the *p*‐value was greater than 0.05, then the null hypothesis was accepted.

OLS regression gives equal weight to all observations, but when homoskedasticity is not present, the cases with larger disturbances have more “pull” than other observations. In this case, a weighted least‐squares regression would be more appropriate, as it downweights those observations with larger variance.

A more serious problem associated with heteroskedasticity (absence of homoskedasticity) is the fact that the standard errors are biased. This leads to incorrect conclusions about the significance of the regression coefficients. Weighted least‐squares regression also addresses this concern but requires a number of additional assumptions.

So, after having verified that the residuals were normally distributed, the assumption of homoskedasticity was also verified in order to select the appropriate iteration algorithm. To that end, the Levene's test was used to test whether the samples had equal variances. For each model, the residual values were divided into three parts with the same number of elements.

If the resulting *p*‐value of Levene's test was less than the significance level (0.05), the obtained differences in residual variances were unlikely to have occurred based on random sampling from a population with equal variances. Thus, the null hypothesis of equal variances was rejected, and it was concluded that there was a difference between the variances in the population.

After having corroborated the requirements of normality and homoskedasticity of the residuals, individual significance tests were analyzed for each parameter, in every model. The significance of a coefficient in a regression model was determined by dividing the estimated coefficient by the standard deviation of this estimate. For statistical significance, we expected that $|t_{\mathrm{ratio}}|>t_{1-\frac{\alpha }{2};n-k}$, (it approached 1.96 as *n* tended to infinity for *α* = 0.05) where *n* was the sample data (in this case 24) and *k* was the number of parameters estimated in the model. Also, we expected the *p*‐value to be less than the significance level (*α* = 0.01 or 0.05). Dependency is a relation between parameters.

If the regression coefficients were significant, the model was accepted. Then, goodness of fit was assessed on comparing the following statistical values: Reduced chi‐square ($\chi_{\nu }^{2}$), adj. *R*‐square ($R_{\mathrm{adj}}^{2}$), standard error of the estimate (SEE), and mean relative deviation (MRD).

Normal probability plots of the residuals were used to check whether the variance was normally distributed as well. If the resulting plot was approximately linear, the error terms were normally distributed. These plots were based on the percentiles versus the residuals, and the percentiles were estimated by

(9) $$\text{Percentile}=\frac{i-\frac{3}{8}}{n+\frac{1}{4}}$$

## 5 RESULTS AND DISCUSSION

### 5.1 Drying curve description

**Figure 1:**
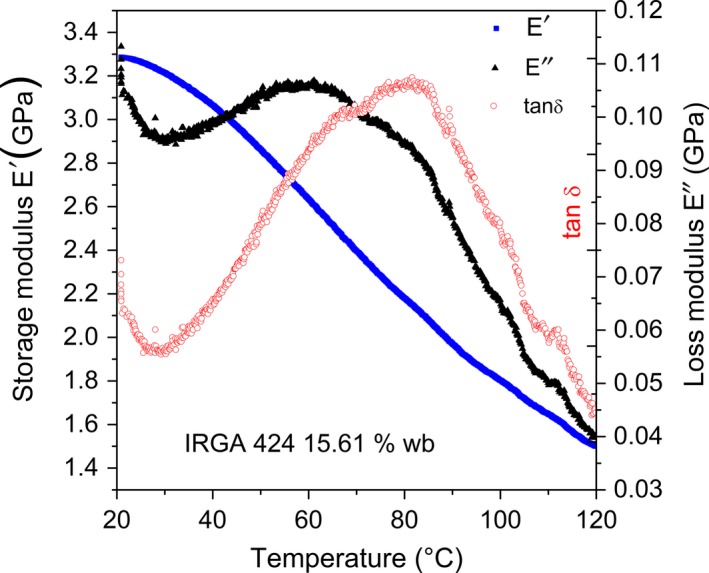
Storage modulus *E′*, loss modulus *E″*, and *tan (*δ*)* as a function of temperature, in degree Celsius, °C of samples of rice flour IRGA 424 with MC of 15.61% w.b.

Figure 1 depicts storage modulus *E′*, loss modulus *E″,* and *tan (*δ*)* as a function of temperature (°C) of samples of rice flour IRGA 424 with MC of 15.61%.

It was clearly identified from the DMA results that Tg was accompanied by a significant loss in *E′*. The storage modulus continuously decreased from the beginning of the heating till the end of the experiment.

The behavior of the *tan (*δ*)* of rice flour as a function of temperature was similar to that of the loss modulus of rice flour as a function of temperature. Both differ from the storage modulus, because they do not decrease consistently across the range, but instead, their values first grow and then decrease, forming a curve with a maximum value.

It can be observed from Figure 1 that the mechanical energy was predominant until approximately 42°C (*E′ *> *E*″), and thereafter, the viscous response became dominant (*E*″ > *E*′). At the beginning of the experiment, between 20 and 30°C, the *tan (*δ*)* and the loss modulus *E*″ showed an initial decrease. Thereafter, both properties increased progressively, and after reaching a peak, it decreased again. Both curves showed an inflection point, reflecting the beginning of the molecular motion according to the volume theory. At this point, the amorphous starch transitioned reversibly from a glassy state into a rubbery state. The *tan (*δ*)* peak can be considered as the end of the glass transition process. As previously mentioned, the glass transition occurred in a range of temperatures.

All the DMA curves obtained from the measurement of the flour samples in the moisture content range analyzed in this study could be described in a similar way.

### 5.2 Tg determination

In this work, Tg (Tg_onset_) was not measured directly as it was not possible to identify clearly the shift or change in slope in the *E′* curve or in the derivative of *E′* curve as a function of temperature. Therefore, Tg was calculated using the other two criteria: the peak of the loss modulus signal (Tg_midpoint_) and the peak of the *tan (*δ*)* (tangent delta) signal (Tg_endset_), as both peaks were sufficiently well defined to calculate the maxima. For each test, the data points around the peak were fitted to a parabola. The maximum was taken as the highest point of the fitted parabola, smoothing out the effects of experimental errors (Pereira & Oliveira, 2000).

Tg values determined in this study for each level of moisture content with their corresponding statistical parameters are presented in Table 1. The SD and CV found were consistent with the class interval chosen to represent the *MC* values of the samples in all cases. The average values found for the Tg_midpoint_ rose from 34.49 to 74.70°C, while those for Tg_endset_ rose from 43.05 to 103.85°C when *MC* decreased 21.68% to 9.71%. Table 1 shows clearly that the Tg decreased with increasing moisture content.

### 5.3 Mathematical models for prediction of Tg

One of the main objectives of this study was to find a mathematical model that allowed us to predict the glass transition temperatures of the variety of rice most widely used and exported in Argentina. To that end, Tg data were fitted to six mathematical equations. Goodness of fit was based on comparing statistical parameters previously explained. Normality and homoskedasticity of residuals could not be rejected in any model, because the *p*‐values obtained by the Shapiro–Wilk and Levene's test were greater than 0.05 in all cases. Results of these tests are shown in Table 2. The significance of the parameters, k and q, obtained from the aforementioned equations was then evaluated. The regression coefficients and the statistical values of the fitted models are shown in Table 3. The Kwei model was discarded as the *p*‐value corresponding to the significance of the parameter q was greater than the significance level of *α* = 0.05 (0.73 for Tg_midpoint_ and 1 for Tg_endset_).

**Table 2:** Results of Levene's and Shapiro–Wilk tests. At the 0.05 level, the data were significantly drawn from a normally distributed population

Model Name	Tg	*p*‐Value	
		Levene's Test	Shapiro–Wilk Test
Jenkel	Midpoint	0.22	0.49
Gordon and Taylor		0.29	0.99
Linearized Gordon and Taylor		0.19	0.92
Kwei		0.31	0.22
New equation		0.32	0.30
Jenkel	Endset	0.23	0.45
Gordon and Taylor		0.38	0.60
Linearized Gordon and Taylor		0.10	0.41
Kwei		0.23	0.45
New equation		0.20	0.40

**Table 3:** The regression coefficients and the statistical values of the fitted models

Tg	Model Name	Properties of regression coefficients						$\chi_{\nu }^{2}$	$R_{\mathrm{adj}}^{2}$	SEE	MRD
		Name	Value	*SD*	*t*‐Value	*p* > |*t*|	DEP				
Midpoint	Gordon Taylor	k	0.35	0.01	66.30	0.00	0.00	24.18	0.91	4.92	8.28
	Kwei	k	0.17	0.02	8.43	0.00	0.97	17.29	0.93	4.16	6.72
		q	11.60	33.40	0.35	0.73	0.97				
	Jenkel	k	−417.32	10.20	−40.93	0.00	0.00	47.03	0.82	6.86	11.52
	Linear Gordon Taylor	k	0.35	0.01	66.78	0.00	0.00		0.99	5.02	3.83
	NE	a	160.27	2.88	5.58	0.00	0.91	17.23	0.93	17.20	3.83
		k	−5.38	0.36	−14.87	0.00	0.91				
Endset	Gordon Taylor	k	0.42	0.01	46.84	0.00	0.00	43.04	0.89	6.56	9.26
	Kwei	k	1.00	411.19	0.00	1.00	1.00	41.16	0.90	6.42	9.2
		q	−329.81	64928.28	−0.01	1.00	1.00				
	Jenkel		−329.84	9.33	−35.35	0.00	0.00	39.37	0.90	6.27	9.2
	Linear Gordon Taylor	k	0.37	0.01	68.72	0.00	0.00		0.99	5.36	3.28
	NE	a	183.95	4.20	43.8	0.00	0.90	41.7	0.89	42.45	8.74
		k	4.05	0.45	−8.9	9.26E−12	0.90				

Note

$\chi_{\nu }^{2}$: reduced chi‐squared; $R_{\mathrm{adj}}^{2}$: adjusted chi‐squared; NE: new equation; SEE: standard error of the estimate; MRD: mean relative deviation, DEP: dependency.

The Fox equation was also discarded as it did not fit the data. This is logical, because this expression can only be applied to components with similar structure, solubility, and physical parameters (cohesive energy density), that is, to mixtures of components with very weak or no specific intermolecular interaction. As the main components of rice are water and starch, their physical parameters (such as thermal capacities) are dissimilar, and thus, there is strong interaction between them.

With the other models whose estimators were significant, the remaining statistics were calculated in order to compare and select the most appropriate mathematical model to predict Tg. The linearized Gordon–Taylor equation was the most appropriate to predict Tg from the experimental data, as it showed the highest values of adj. *R*‐square and the lowest values of SEE and MRD.

The second place in the ranking of models was occupied by the Gordon–Taylor equation for Tg_midpoint_ and Jenkel for *T*g_endset_, applying the same criteria. Note that these models were fitted based on the measured data in the range of moisture content 9.7%–21.7% wet basis.

**Figure 2:**
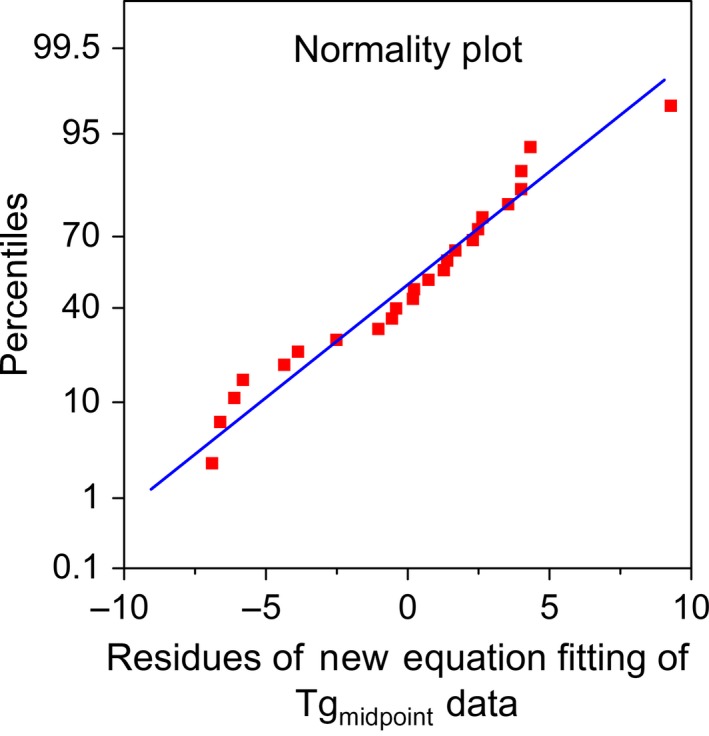
Normality plot (percentiles vs. residues) of the new equation

Equation (8) is a better alternative to the Gordon–Taylor equation to predict Tg_midpoint_ as it has higher values of adj. *R*‐square and lower value of MRD. This equation is comparable to the Jenkel equation for Tg_endset_ prediction, as it presented similar statistical parameters. Figure 2 shows that the residuals of this equation were normally distributed. It should be mentioned that final expressions of these models cannot be used to estimate Tg of samples or rice beyond the MC range used in this study.

**Figure 3:**
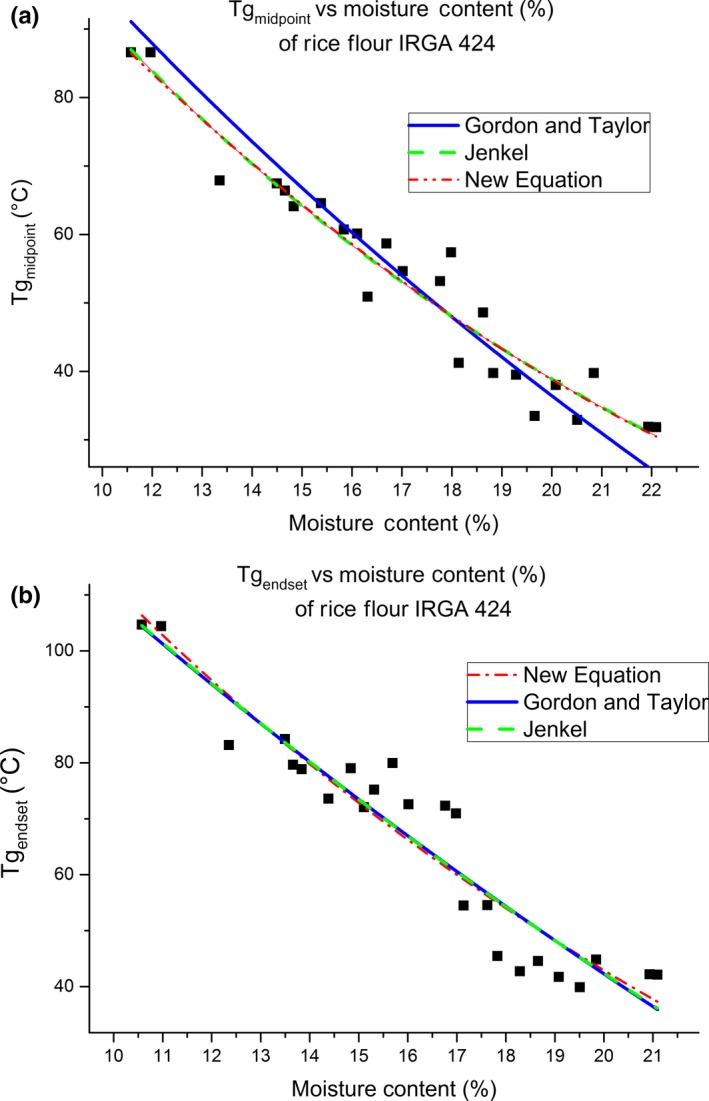
The dependence of Tg on moisture content (% w.b.) of samples of rice IRGA 424, as well as the fitted models. (a) Tg_midpoint_ and (b) Tg_endset_

Figure 3 presents the dependence of Tg on MC as well as the fitted models. It shows clearly that water acted as a strong plasticizer enhancing molecular mobility resulting in lower Tg according to the free volume theory. Tg values did not decrease sharply at higher moisture content, due to the limit of the plasticization effect of water on rice. In this case, additional water did not interact strongly with the starch or protein molecules and therefore failed to decrease the Tg rapidly. The system behaved as phase separated into water and solid.

### 5.4 Comparison with literature data

**Figure 4:**
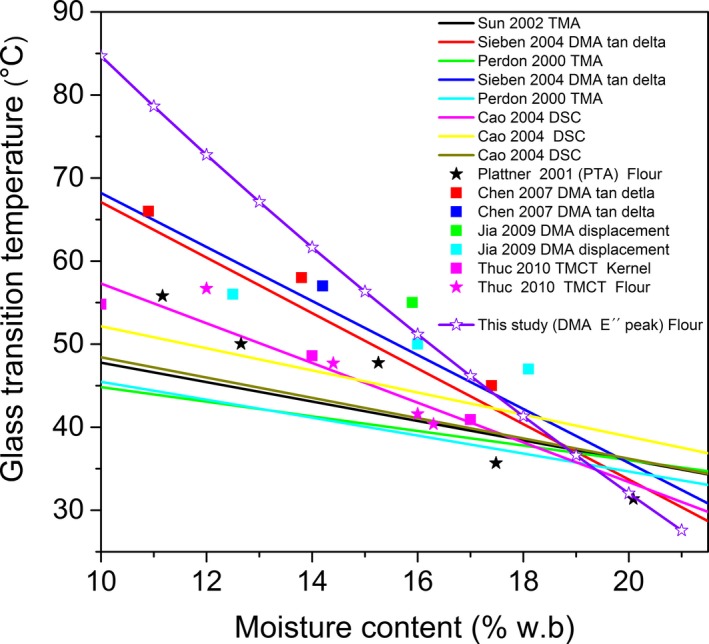
Comparison between reported Tg values from the literature and Tg_midpoint_ values obtained in this study

Tg of IRGA 424 flour was compared with the literature data in which Tg was obtained by different techniques (TMA, DSC, DMA, TMCT, phase transition analysis [PTA]). The data compilation is listed in Table 4 and shown in Figure 4. Some authors provided correlations of Tg versus *MC*, while others reported Tg values at a specific moisture content.

**Table 4:** Published data on Tg of rice kernel and flour as compared to Tg rice flour

Reference	Method	Samples	Variety	*M*% w.b.	Tg	*R* ^2^
This study	DMA Single cantilever bending	Brown flour	IRGA 424 LG	10–22	$T_{g}=a-1,000\frac{\left(e^{kw_{1}}-1\right)}{k}$ a=160.27k=−5.38	0.93
Cao et al. (2004)	DSC	Brown kernel	Akitakomachi SG	12–25	Tg (°C) = 81.19 − 2.39 *M*	0.93
			L201 LG	12–25	Tg (°C) = 65.46 − 1.33 *M*	0.92
			Delta LG	12‐25	Tg (°C) = 60.62 − 1.22 *M*	0.69
Chen et al. (2007)	DMA Parallel Compression mode	Brown kernel	Jing Rice No.3 SG	14.2	57	
			FuFengYou 11 LG	10.9, 13.8, 17.4	66, 58, 45	
Jia et al. (2009)	DMA Parallel Compression mode	Brown kernel	LiaoJing SG	16.0	50	
			R894 MG	12.5, 16, 18.1	56, 50, 47	
			IR‐2 LG	15.9	55	
Siebenmorgen et al., 2004;	DMTA Parallel Compression mode	Brown kernel	Drew LG	7–22	Tg (°C) = 100.5 − 3.34 *M*	0.81
			Bengal MG	7‐22	Tg (°C) = 100.7 − 3.25 *M*	0.82
Sun et al. (2002)	TMA	Brownkernel	Drew LG	7.6‐21.7	Tg (°C) = 59.47 − 1.17 *M*	0.57
Perdon et al., 2000;	TMA	Brown kernel	Bengal MG	3–27	Tg (°C) = 53.63 − 0.88 *M*	0.54
		Brown kernel	Cypress LG	3–27	Tg (°C) = 56.27 − 1.08 *M*	0.38
Plattner et al. (2001)	PTA	Flour	a	a	a	a
Thuc et al. (2010)	TMCT	Brown flour	YRM64 LG	12,14.4, 16, 16.3	56.7, 47.7, 41.6, 40.38	
		Brown kernel	YRM64 LG	10, 14, 17	54.8, 48.6, 40.9.	
Talab et al. (2012)	DSC	Brown kernel	MR219	7.4–26.8	9.65–61.79	a
Nithya 2014	PTA	Roasted flour	Bengal MG	9–27	160–80	a

Notes

TMCT: thermal mechanical compression test, DSC: differential scanning calorimetry; MDSC: modulated differential scanning calorimetry; TMA: thermomechanical analysis; DMTA: dynamic mechanical thermal analysis; PTA: phase transition analyzer; SG: short grain; MG: medium grain; LG: long grain; NR: not reported.

a Values plotted in Figure 4 were taken from Siebenmorgen et al. (2004).

At high moisture content, the midpoint and the endset of the glass transition tended to coincide. Differences between Tg_midpoint_ and Tg_endset_ with the same order of magnitudes have also reported by other authors. Pereira and Oliveira (2000) made glass transition measurements for native and pregelatinized wheat flour pellets. They reported a clear change of their viscoelastic properties according to the expected glass transition effects: a drop of *E′* around 55°C, a peak of *E″* around 65°C, and a peak of *tan (*δ*)* around 85°C. These authors pointed out that the endset of glass transition (taken as the peak in *tan* (δ)) was significantly higher for native flour samples of wheat. They supposed that this may be caused by the larger heterogeneity of the molecular arrangements as well as differences in actual water content.

Rahman, Al‐Marhubi, and Al‐Mahrouqi (2007) measured glass transition of spaghetti by DMA from the change in slope in *E′*, which was 10.5°C lower than the value measured by *tan (*δ*)* peak. They found it difficult to find a maximum in the loss modulus *E″* curve.

Among the researchers that used DMA, Chen et al. (2007) determined Tg from *tan (*δ*)* peak, Siebenmorgen et al. (2004) from the change in the slope of *tan (*δ*)* curve and related this value to the onset of glass transition and Jia et al. (2009) from the change in the slope of the displacement curve as a function of temperature.

Tg_midpoint_ temperatures of IRGA 424 were comparable to reported values (Figure 4), especially those given by Chen et al. (2007) and Jia et al. (2009), while Tg_endset_ corresponding to the *tan (*δ*)* peak signal turned out to be significantly higher. At 20% MC Tg literature data ranged from 31°C (Plattner, Strahm, & Rausch, 2001) to 38.9°C (Cao, Nishiyama, & Koide, 2004). Tg_endset_ of IRGA 424 with 19.8% MC resulted 43.16°C (7% higher than the upper limit). At 13% MC compiled Tg_endset_ values ranged from 42°C (Perdon, 1999) to 58°C (Siebenmorgen et al., 2004), while Tg_endset_ of IRGA 424 reached 82.35°C (41% higher than the upper limit).

Previous studies have concluded that in the case of rice, Tg is primarily influenced by its starch content. Tg values of rice flour measured by Plattner et al. (2001) with a phase transition analyzer were comparable to the Tg values of brown rice kernels measured with DMA by Jindal and Siebenmorgen (1994). Thuc et al. (2010) applied independent tests with the same procedure to rice flour and to individual rice kernels to determine the Tg in order to investigate whether single rice kernel can be used directly instead of ground flour. They found that the measured Tg was almost identical for both individual rice kernel and rice flour at the same moisture content. This indicates that the Tg values determined in rice flour samples of IRGA 424 can be applied to analyze drying conditions of rice kernels incorporating glass transition principles.

## 6 CONCLUSIONS

Tg data of rice flour samples of IRGA 424 at different *MC* were determined satisfactorily by DMA. The relaxation phenomenon of rice flour samples was much more obvious in the E″ or *tan (*δ*)* plots than in the storage modulus curve. This is why Tg was calculated using the peak of the loss modulus signal (Tg_midpoint_) and the peak of the tangent delta signal (Tg_endset_). At higher moisture content, these peaks tended to coincide. Tg_midpoint_ was comparable to the published data, while Tg_endset_ turned out to be higher. In the present study, the temperature read at *tan (*δ*)* peak was better defined, but for rice drying application processes, Tg_midpoint_ is more appropriate to be considered as a reference. Water acted as an effective plasticizer reducing the Tg in the samples of rice flour. The values found for the IRGA 424 variety rose from 42.13 to 104.69°C as moisture content decreased from 22.3% to 9.3%. To relate Tg to *MC*, the best fit was obtained with the linearized Gordon–Taylor equation. However, the new equation presented in this work was proposed as a good alternative to predict Tg of IRGA 424, without the need to perform a prior linearization.

## CONFLICT OF INTEREST

The authors state that there were no conflict of interests.

## ETHICAL STATEMENT

This study does not involve any human or animal testing.
